# Comparative homegarden medical ethnobotany of Naxi healers and farmers in Northwestern Yunnan, China

**DOI:** 10.1186/1746-4269-10-6

**Published:** 2014-01-10

**Authors:** Lixin Yang, Selena Ahmed, John Richard Stepp, Kai Mi, Yanqiang Zhao, Junzeng Ma, Chen Liang, Shengji Pei, Huyin Huai, Gang Xu, Alan C Hamilton, Zhi-wei Yang, Dayuan Xue

**Affiliations:** 1 College of Life and Environmental Science, Minzu University of China, 27 Zhong-Guan-Cun South Avenue, Beijing 100086, China; 2 Kunming Institute of Botany, Chinese Academy of Sciences, Kunming 650201, China; 3 Sustainable Food and Bioenergy Systems Program, Department of Health and Human Development, Montana State University, Bozeman, MT 59717, USA; 4 Department of Anthropology, Ethnobiology Laboratory, University of Florida, P.O. Box 117305, Gainesville, FL 32611-7305, USA; 5 College of Forestry and Vocational Technology in Yunnan, Kunming 650224, China; 6 Southwest Forestry University, Bailongshi, Kunming 650224, China; 7 College of Bioscience and Biotechnology, Yangzhou University, Yang Zhou 225009, China; 8 128 Busbridge Lane, Godalming, Surrey GU 7 I QJ, UK

**Keywords:** Homegardens, Medicinal plants, Naxi, Ethnomedicine, Healers

## Abstract

**Background:**

Homegardens are ecologically and culturally important systems for cultivating medicinal plants for wellbeing by healers and farmers in Naxi communities of the Sino Himalayan region. The cultivation of medicinal plants in Naxi communities and associated ethnomedical knowledge base for maintaining and utilizing these resources is at risk with expanded commercialization of natural resources, development policies and rapid socio-economic change in China. Research is needed to understand the medicinal plant species maintained in Naxi homegardens, their use and contribution to community wellbeing, and how these practices and knowledge base varies between Naxi healers and farmers in order to develop plans for biodiversity conservation and preservation of ethnomedical practices. The main objective of this study is to document and compare medicinal plant species in Naxi homegardens and associated ethnomedical knowledge between Naxi healers and farmers.

**Methods:**

Ethnobotanical homegarden surveys were conducted with three Naxi healers and 28 farmer households in two Naxi communities in Lijiang Prefecture in Northwest Yunnan Province of China. Surveys included inventories of medicinal plants in homegardens and semi-structured interviews with homegarden managers to document traditional medicinal uses of inventoried plants. Inventoried plants were classified into 13 ‘usage categories’ of medical condition groupings that impact a system of the body. Finally, plant species richness was calculated for each homegarden and species richness was compared between healers and farmers as well as between study sites using a Least Square Means Tukey HSD function.

**Results:**

Ethnobotanical surveys at the study sites found that 13% of households rely exclusively on traditional Naxi medicine, 26% exclusively use Western medicine and 61% use a combination of traditional Naxi and Western medicine. A total of 106 medicinal plants were inventoried in Naxi homegardens representing 50 botanical families. Over 85% of inventoried medicinal plants were herbaceous. The most represented families were Asteraceae (12.8%), Ranunculaceae (8.3%), Apiaceae (8.3%), and Polygonaceae (7.3%). The primary medical functions of inventoried plants were to treat inflammation (73 species), circulatory system disorders (62), nervous system disorders (41), detoxification (39), digestive system disorders (33), muscular-skeletal system disorders (26), genitourinary system disorders (26), skin conditions (23), respiratory systems disorders (22), and cold and flu (20). Local herbal experts maintained greater medicinal plant species richness in their homegardens compared to local farmers as well as had greater knowledge of medicinal functions of plants. Healers maintained medicinal plants primarily for healing while farmer households maintained approximately 90% of the medicinal plants in their homegardens for commercialization and the remaining for household healthcare.

**Conclusions:**

This study highlights the importance of biodiversity and traditional ecological and medical knowledge for human wellbeing and livelihoods in Naxi communities. Conservation efforts and policies are necessary to preserve the ecological and cultural base that maintains medicinal plant use by both healers and farmers in Naxi homegardens of the Sino Himalayan region.

## Background

Communities worldwide manage medicinal plants in homegardens to support wellbeing and livelihoods [1-10]. Studies that indicate the reliance of communities on plants growing in disturbed and anthropogenic spaces, coupled with studies showing higher use values for cultivated food and medicinal plants compared to wild plants, emphasize the importance of investigating homegardens in the provision of medicinal plant remedies [11-17]. Anthropogenic environments such as homegardens are accessible systems for communities to manage, utilize and transmit ecological and ethnomedical knowledge to support household wellbeing [18-25].

The ethnomedical systems of China’s numerous socio-linguistic groups play a crucial role for community health in the country’s indigenous areas, many of which are located in habitats of high biodiversity. China’s 55 minority socio-linguistic groups are recognized to utilize more species of medicinal plants compared to the Traditional Chinese Medicine (TCM) system of the dominant Han population [26]. There have been approximately 7,000-8,000 documented species found in the ethnobotanical literature of China’s minority communities and 4,758 species in TCM [26]. The gathering of medicinal plants also provides an important livelihood activity for many indigenous communities in China.

China’s Northwest Yunnan Province is recognized for its rich biodiversity and cultural history associated with the management and utilization of medicinal plants. NW Yunnan is located in the Three Parallel Rivers region and is listed as a World Natural and Cultural Heritage Site by UNESCO, making it global priority site for biodiversity conservation. The region’s exceptional altitudinal range, topography and climatic variability have fostered centers of plant species endemism [27,28]. NW Yunnan harbors over 3,500 endemic plant species, many of which are utilized by local communities including the Naxi [29,30].

**Figure 1:**
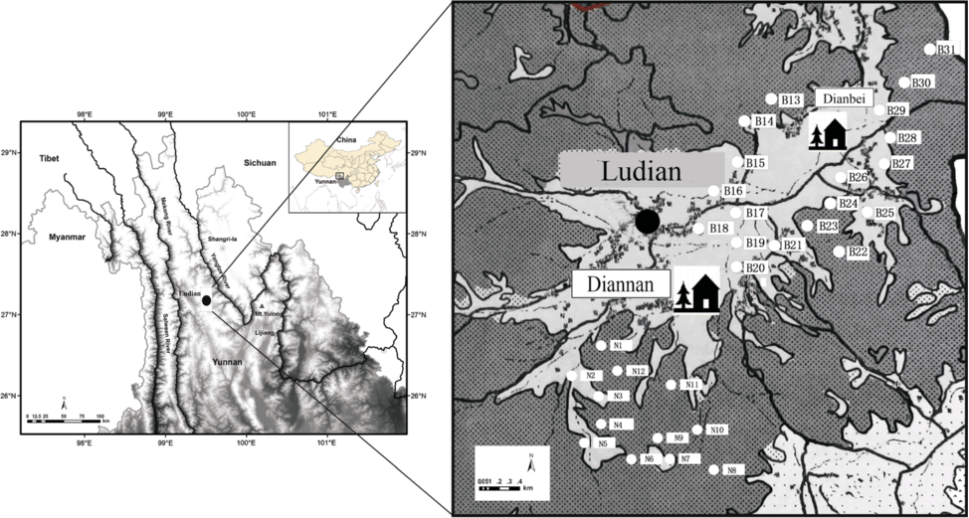
Map of Northwest Yunnan, China.

**Figure 2:**
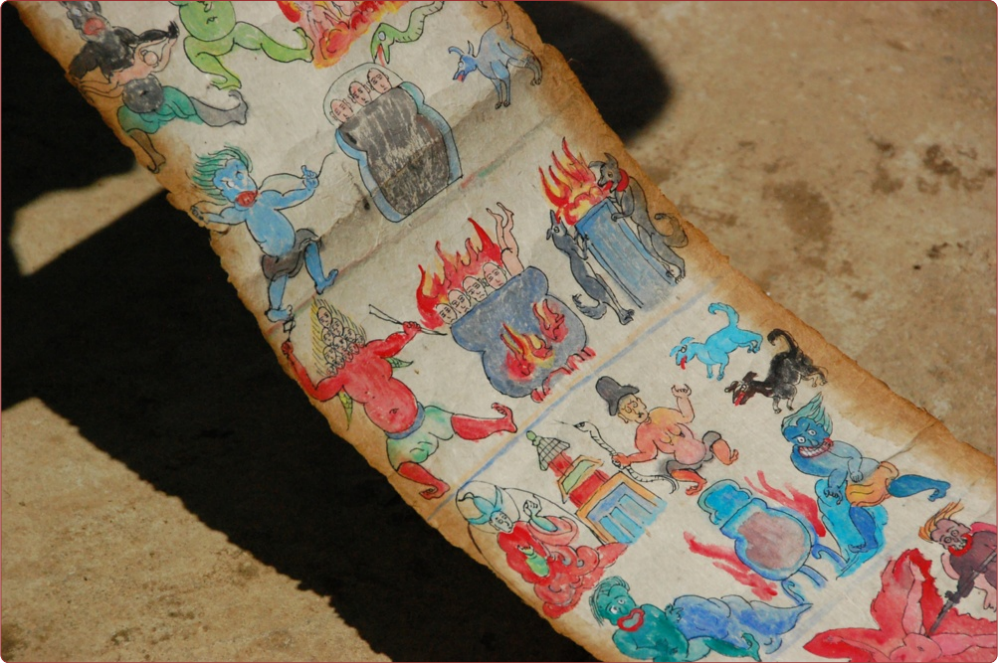
**Dongba scroll containing the world's last remaining pictographic writing system.** (Photo by J.R. Stepp).

The Naxi are a Burmo-Naxi-Lolo sociolinguistic sub-group of the Tibeto-Burman group within the Sino-Tibetan family [31]. They primarily inhabit the highlands of Lijiang Naxi Autonomous Region in the eastern Himalaya of China’s Northwest Yunnan Province with a population of approximately 300,000 (Figure 1). Historically, the Naxi relied on an indigenous system of Bon practice to treat health conditions primarily through consultation with local shaman priests known as Dongba (Dto’mba) as well as through herbal healers and self-care [32,33]. The traditional Bon practice is founded on animist and shamanistic traditions with links to pre-Buddhist and Buddhist Tibetan practice and many of these traditions remain through the world's only remaining pictographic writing system (Figure 2).

The Naxi, like many indigenous groups in Yunnan, China, have a long history and traditional knowledge of growing food and medicinal plants in gardens adjacent to their homes to support their livelihoods [34-37]. Previous research has found that over 60% of Naxi farmers in Ludian Township of NW Yunnan cultivate medicinal plants for household and commercial use [38]. In addition, approximately 30% of all medicinal plants used in Naxi medicine by traditional healers are from homegardens, 60% from wild habitats, and 10% from other localities [39]. This study builds on previous ethnobotanical work by focusing on plant species and their medicinal functions found in Naxi homegardens in China’s Northwest Yunnan Province. The present study compares homegardens managed by Naxi healers and farmers to understand variation between medicinal practitioner experts and common households. This study provides a useful baseline for future study on medicinal plant species richness, usage and natural product discovery in the Sino - Himalayan region.

## Methods

### Study sites

Research was conducted in Ludian Township of Yulong County of Lijiang City in China’s Northwest Yunann Province, an area identified by the Yunnan Provincial Scientific and Technological Department as the “home of medicinal plants” in provincial history and tradition. Two study sites communities were selected for this study: Diannan (甸南 translates to “grassy marshland in the south”; Northern latitude 27°12′52〞and Eastern latitude 99°28′57〞), and Dianbei (甸北 translates to “grassy marshland in the north”; Northern latitude 27°11′26〞 and Eastern latitude 99°27′34〞). Study sites were selected after consulting local government officials and preliminary field visits to identify villages where traditional ethnomedical practice and knowledge seemed most intact as well as areas that housed practicing traditional healers. Dianbei village had one healer at the time of research and Diannan village had two healers at the time of research.

The study sites are situated within the Hengduan Mountains at the eastern end of the Himalayas, southeast of the Tibetan Plateau and midstream of the Jinsha River. Lijiang has the climate of the Hengduan Mountain valley as well as that of Northwest Yunnan Plateau. It has high terrain in the northwest and low terrain in the southeast sloping from 5,596 m down to 1,015 m. This elevational range is accompanied by variation in climate, habitats, soils, and ecotypes that provides diverse conditions for medicinal plants.

The community of Dianbei has 103 households with a population of 318 and the community of Diannan has 140 households with a population of 586. Both villages settlements are located at an altitude between 2,400 m-2,600 m. The distance between the two study sites is 6.5 km. Twelve percent of the total area is under agricultural use) with forest, scrub and pasture compromising the remaining land use. Households follow subsistence livelihood relying on a mosaic of land use in their surroundings coupled with the commercialization of natural resources and off-farm labor.

### Ethnobotanical surveys

Interviews were conducted with a total of three healers and 28 farmer households. We interviewed one healer in Dianbei village and two healers in Diannan village. In addition, we interviewed 11 farmer households in Dianbei village and 16 farmer households in Diannan village. We selected to interview the primary person managing each homegarden and responsible for collecting medicinal plants when a household member gets ill. All informants were over 40 years old with an average age of 52 years. Ten of the informants were female and the remaining 21 informants were male.

Semi-structured interviews included documentation of local names, plant parts, preparation methods, mode of administration, health functions of medicinal plants found in homegardens and percentage of household income derived from the sale of medicinal plants in farming land. Voucher specimens were collected under the guidance of local herbal experts and pressed and dried in the field. Taxonomic plant identification was confirmed at Kunming Institute of Botany, Chinese Academy of Science and specimens were deposited at KIB for long-term storage.

### Medicinal classification of plant use

We followed the medical classification developed by Cook [40] to categorize aliments well known by herbal experts into 13 ‘usage categories’ of medical condition groupings that impact a system of the body [41]. The categories of medical conditions applied include: injuries (INJ), muscular-skeletal system disorders (MUS), infections/infestations (INF2), digestive system disorders (DIG), skin-subcutaneous cellular tissue disorders (SKI), pregnancy/birth/puerperium disorders (PRE), sensory system disorders (SEN), nutritional disorders (NUT), nervous system disorders (NER), genitourinary system disorders/effects (GEN), respiratory system disorders (RES) and circulatory system disorders (CIR). Four usage categories were added to this study that represent Culture-Bound Syndrome (CUL) including children febrile convulsion, Ill-defined Symptoms (IDS) including kidney conditions and fatigue/malaise, inflammation (INF1) and detoxification (DET) as defined in Traditional Chinese Medicine (TCM) and folk medicine systems of China.

### Species richness and comparative ethnobotany

Plant species richness of medicinal plants in each homegarden was determined using Hulbert’s index (1972). A Least Square Means Tukey HSD function was performed using JMP 10.0 (SAS Institute Inc.) to determine how medicinal plant species richness of Naxi homegardens varies with between specialist healer and generalist farmer stakeholders as well as between the two study sites.

## Results

### Ethnobotanical surveys

Ethnobotanical surveys at the study sites found that 13% of informants rely exclusively on traditional Naxi medicine, 26% exclusively use Western medicine available from the local clinic and 61% use a combination of traditional Naxi and Western medicine. Inventories a documented a total of 106 medicinal plants from 50 botanical families used to treat over 160 health conditions. Additional file 1: Table S1 lists ethnobotanical information for each species including scientific name, local names, genera, habitat, plant parts used, description of uses and percent of informants who provided the information.

Findings indicate that over half (58.7%) of documented plants belong to 10 botanical families. The most represented families in the medicinal plant homegarden inventories include Asteraceae (14 species), Ranunculaceae (9), Apiaceae (9), Polygonaceae (8), Labiatae (5), Orchidaceae (4), Liliaceae (4), Rosaceae (4), Campanulaceae (4) and Saxifragaceae (3). The majority of the documented medicinal plants in Naxi homegardens are herbaceous species (87.2%), followed by woody trees (7.3%), shrubs (2.8%), and lianas (1.8%). Roots (45%) were the most frequently used plants parts for medicine accounting for 63 prescriptions, followed by the whole plant (33.9%), combinations of roots and stem (12.8%), fruits (3.7%) and bark (2.8%). Flowers, leaves, sap, and branches accounted for the remaining minor percentages of plant parts. Table 1 shows the most frequently inventoried medicinal plants at the study sites. In addition to medicinal value, most of these plants are also valued for their economic, edible and ornamental values.

**Table 1:** Most frequently inventoried medicinal plants

**Species name**	**Medicinal vale**	**Edible value**	**Economic value**	**Ornamental value**
*Aconitum carmichaeli *Debx.	√	√	√	√
*Aucklandia lappa* Decne.	√	√	√	
*Chaenomeles sinensis* (Thunb.) Makino	√	√	√	
*Fallopia multiflora* (Thunb.) Harald.	√	√	√	√
*Foeniculum vulgare* Mill.	√	√	√	
*Gentiana robusta* King ex Hook.f.	√			√
*Lactuca sativa* L.	√	√	√	
*Ligusticum chuanxiong* S.H.Qiu	√	√		
*Mentha spicata* L.	√	√	√	
*Paeonia delavayi* Franch.var. Lutea (Delav. Ex Franch.) Finet. Et Gagnep.	√		√	√
*Paris polyphylla* Smith var yunnanensis	√		√	√
*Platycodon grandiflorus* (Jacq.) A.DC.	√	√	√	
*Prinsepia utilis* Role	√	√	√	√
*Zanthoxylum bungenum* Maxim	√	√	√	√

The most frequently inventoried medicinal plants at the study sites are also valued for various other properties by households including edible, economic, and ornamental.

Findings show that the vast majority of inventoried medicinal plants are used in treatment in combination with other plants and a few are used as single plant remedies. The preparations of plants for medicinal treatment include primarily decoction in water for internal consumption. A few preparations also involve infusion with alcohol and honey as a tonic. Some remedies include preparation and consumption of medicinal plants with food items such as with meat, egg, rice, and honey to “strengthen the body”. Some of the remedies are used externally as poultices.

Farmers cultivate medicinal plants in their homegardens for household healthcare as well as for commercial purposes while healers only cultivate medicinal plants in their homegardens for healing purposes and rarely sell species. Approximately 10% of medicinal plants grown by farmers are used for self-care and 90% are sold in herbal markets to generate household income. Farmers do not provide plant material to healers in the community. Table 2 shows the most commonly commercialized medicinal plants grown in homegardens of Diannan and Dianbei villages. Fourteen commercialized medicinal plant species in the seedling stage were cultivated in the homegardens of over half the households at the study sites. Over 80% of households at the study sites derive their income from the commercialization of medicinal plants. Households derive an average of 45% of their income from the commercialization of medicinal plants. In additional to medicinal values, many of these species also have edible, ornamental and economic values for Naxi communities as well are considered to serve an ecological role.

**Table 2:** Commercialized medicinal plant species

**Species name**	**Market price RMB /Kg**	**Plant part traded**	**Dry or fresh plant traded**	**Degree of commercialization**
*Aucklandia lappa* Dencne	16	Root	Dried	Medium
*Gastrodia elata* Bl.	400	Root	Dried	Medium
*Gentiana robusta* King ex Hook. F	16	Root	Dried	High
*Platycodon grandiflorum* (Jacq.) A.DC.	30	Root	Dried	High
*Ligusticum sinense* cv. Chuanhsiung Shan ex k. C. Fu	18	Root	Dried	High
*Rheum palmatum* L	8	Root	Dried	Low
*Notopterygium franchetii* Boiss	24	Root	Dried	Low
*Dipsacus mitis* D. Don	7	Root	Dried	Medium
*Atractylodis macrocephalae* Koidz.	16	Root	Dried	Medium
*Paris polyphylla Smith var yunnanensis* (Fr.) Hand.-Mazz.	200	Root	Dried	High
*Cynanchum otophyllum* Schneid.	400	Root	Dried	Medium
*Aconitum carmichaeli* Debx.	8	Root	Fresh	Low
*Bletilla sinensis* (Rolf) Schltr.	28	Root	Dried	Low
*Ligusticum chuanxiong* S.H.Qiu	14	Root	Dried	Medium

Over half the study site households were found to maintain fourteen commercialized species.

### Medicinal classification of plant use

Compiled findings from both study sites show that the most frequently reported health conditions treated by the inventoried medicinal plants are inflammation (73), circulatory system disorders (62), nervous system disorders (41), detoxification (39), digestive system disorders (33), muscular-skeletal system disorders (26), genitourinary system disorders (26), skin conditions (23), respiratory systems disorders (22), and cold and flu (20). The most frequently reported health conditions treated by the inventoried medicinal plants by healers in Dianbei village are cold and flu, rheumatism, digestive system disorders, genitourinary system disorders, pain, circulatory system disorders, muscular-skeletal system disorders, skin conditions, nervous system disorders, and inflammation. The most frequently recorded health conditions for medicinal plant use by farmers in Dianbei village are circulatory system disorders, respiratory system disorders, colds and flu, nutritional disorders, and inflammation. In Diannan village, the most frequently reported health conditions treated by inventoried medicinal plants by healers in Diannan village are inflammation, cold and flu, respiratory system disorders, genitourinary system disorders, digestive systems disorders, detoxification, muscular-skeletal system disorders, injuries, and skin conditions. The most frequently mentioned health conditions treated by the inventoried medicinal plants by farmer households in Diannan are digestive system disorders, nutritional disorders, cold and flu, inflammation and circulatory system disorders.

### Plant species richness and comparative ethnobotany

Species richness of medicinal plants in the homegardens of healers was found to be significantly higher than that of farmers at the two study sites (p > 0.048). Least Square Means of medicinal plant species richness maintained by healers was 0.347 and that of farmers was 0.161. No significant difference was found in overall species richness of medicinal plants in the homegardens of the two study sites with overall medicinal plant species richness in Diannan village having a Least Square Means of 0.284 and Dianbei having a Least Square Means of 0.225. In Dianbei village, 52 medicinal plants from 32 botanical families were inventoried in homegardens managed by healers and a total of 24 medicinal plants from 21 botanical families were inventoried in homegardens managed by farmers. In Diannan village, a total of 39 medicinal plants from 22 botanical families were inventoried in homegardens managed by healers and a total of 26 medicinal plants from 13 botanical families were inventoried in homegardens managed by farmers.

## Discussion

This study highlights the rich biodiversity of medicinal plant cultivation and ethnomedical practice in homegardens of Naxi communities to maintain wellbeing and to support livelihoods. Findings show that Naxi communities manage plant diversity in their homegardens to treat a wide range of health conditions that inflict local households. A total of 106 plant species were inventoried from 50 botanical families to treat 160 health conditions. Plant species maintained by healers was found to be significantly distinct than plant species managed by farmers while no significant difference was found in medicinal plant species richness cultivated at the two study sites. Ethnomedical usage of plants managed by healers was markedly distinct from usage categories managed by farmers. Findings show that plant resources are important to community wellbeing at the study sites with the majority of households relying on an integration of Naxi and Western medicine for community wellbeing. The minority of households that rely solely on Naxi medicine indicates that cultural efforts may be needed in the future for the preservation of traditional Naxi medicine with further socio-economic change at the study sites. Economic incentives provided by medicinal plant markets helps ensure that these resources will continue to be cultivated at the study sites.

The present study contributes to the literature on Naxi ethnomedicine in the Sino-Himalayan area. Previous ethnomedicinal work in the Sino-Himalayan area shows similar results on the most frequently prevalent plants families used for medicine. While households manage a range of medicinal plants from over 100 botanical families, over half the plants belong to 10 botanical families with Asteraceae being the most prevalent. Nine of these prevalent botanical families have been previously reported to be most prominent in Naxi ethnomedical systems [32,33] including Asteraceae, Labiatae, Ranunculaceae, Apiacea, Rosaceae, Liliaceae, Polygonaceae, Orchidaceae, and Campanulaceae. The finding that the majority of inventoried medicinal plants in Naxi homegardens are herbaceous species supports previous ethnobotanical findings [16] and highlights the importance of conserving herbaceous plant diversity for community wellbeing.

Inflammation and circulatory system disorders were found to be the most reported use categories for medicinal plants maintained in Naxi homegardens. Findings indicate that healers and farmers maintain medicinal plants in their homegardens to treat distinct health conditions. The most prevalent health conditions for inventoried medicinal plants managed by healers were for circulatory system disorders, traumatic injury, respiratory system conditions, digestive system disorder (DIG), strengthening bones and tendons (MUS), and genitourinary system disorders (GEN). Alternatively, the medicinal plants maintained by local farmers include remedies for common health conditions and for commercialization. Previous ethnomedicinal work in the Sino-Himalayan area shows similar results on the most frequently mentioned health conditions for inventoried medicinal plants including for inflammation, digestive system disorders, injuries and bruises, rheumatism, respiratory system disorders and dermatological illnesses [42-45].

Findings that healers manage greater numbers of medicinal plants compared to local farmers emphasizes the role of local healers as medical specialists in Naxi communities and demonstrates their ethnomedical knowledge. Farmers have knowledge of common illnesses that they can treat through self-care and indicates their generalist knowledge. It is important to recognize the distinct diversity and plant use maintained by these two stakeholder groups for comprehensive conservation and cultural revitalization efforts. While significant differences were found between healers and farmers in medicinal plant species richness maintained in homegardens, there was no significant difference between the two study site communities. These findings demonstrate cultural consensus of Naxi ethnomedicial systems across the study sites in terms of species richness. The greatest agreement on inventoried medicinal plants and their uses between the study sites was found for highly commercialized species.

Commercialization of medicinal plants for China’s growing herbal market constitutes the majority of farmer income at the study sites and is a major driver of the persistence of medicinal plants in Naxi communities. As new medicinal plant products are developed for commercialization on the basis of the Naxi ethnomedical system, it is expected that local value systems and medicinal plant composition of homegardens will shift with adaptation to meet market needs. Efforts are needed to ensure that economic incentives are provided to maintain species richness of medicinal plants at the study sites as well as their associated ethnomedical knowledge.

## Conclusions

Traditional ethnomedical systems comprising of medicinal plants persist in integration with Western medicine in Naxi communities in the Sino-Himalayan area. Both expert healers and generalist farmer households support the traditional Naxi ethnomedical system through cultivation of medicinal plants in their homegardens to treat a wide range of health conditions. However, healers and farmers maintain distinct species in their homegardens for distinct health conditions with inflammation being the most frequently reported health condition for medicinal plant use. Farmers manage medicinal plants to treat more common conditions compared to healers. In addition, approximately 90% of the medicinal plant resource base managed by farmers is for commercialization for China’s growing herbal market, highlighting the role of markets in providing economic incentives for *in situ* biodiversity conservation and ethnomedical preservation.

Naxi homegardens provide *in situ* conservation spaces for medicinal plant germplasm as well as a shelter for native, rare and endangered plants. Medicinal plants maintained within Naxi homegardens are valued for diverse properties including edible, economic and ornamental properties. In addition, Naxi homegardens provide important habitats for the introduction and domestication for wild medicinal plants and as a nursery for plant propagation. These ecologically and culturally important spaces for the transmission and preservation of ethnomedical knowledge that support community wellbeing and livelihoods are at risk with rapid socio-economic, policy, land use and environmental changes in China. Conservation efforts and evidence-based policies are necessary to preserve the ecological and cultural base that maintains medicinal plant use and community wellbeing by both healers and farmers in Naxi homegardens. Economic incentives provided by markets are one way to ensure the protection of ethnomedical plant knowledge in Naxi communities. Future studies can shed insight on the success of such policies and market-based conservation efforts.

## Competing interests

The authors declare that they have no competing interests.

## Authors’ contributions

LY conceived of the study, participated in its design, conducted primary data collection and helped to draft the manuscript. JRS and SA contributed to the study design, data analysis and manuscript preparation. KM assisted in the collection of data and fieldwork logistics. YZ assisted in the collection of data and fieldwork logistics. JM assisted in the collection of data and fieldwork logistics. CL assisted in the collection of data and fieldwork logistics. SP, ZY, and ACH assisted in the collection of data and fieldwork logistics. HH assisted in the collection of data and fieldwork logistics. GX assisted in the collection of data and fieldwork logistics. DX provided laboratory space and helped in the analysis of data. All authors read and approved the final manuscript.

## Supplementary Material

Additional file 1: Table S1 Medicinal plants in Naxi homgardens. Naxi communities in Northwest Yunnan cultivate diverse medicinal plants in their homegardens to maintain a wide range of health conditions. Click here for file
