# Supplementary material for: Comparative homegarden medical ethnobotany of Naxi healers and farmers in Northwestern Yunnan, China
Source: J Ethnobiol Ethnomed. 2014 Jan 10;10:6. doi: 10.1186/1746-4269-10-6 (PMC3907136; doi:10.1186/1746-4269-10-6)
Supplement: Additional file 1: Table S1 — Medicinal plants in Naxi homgardens. Naxi communities in Northwest Yunnan cultivate diverse medicinal plants in their homegardens to maintain a wide range of health conditions. [file 1746-4269-10-6-S1.doc]

**Additional file 1: Table S1 Medicinal plants in Naxi homgardens**. Naxi communities in Northwest Yunnan cultivate diverse medicinal plants in their homegardens to maintain a wide range of health conditions.

| **Scientific name** | **Family name** | **Local name** | **Habit** | **Parts used** | **Uses recorded** | **Locations** | **Inf. No.** | **Vou. No.** |
| --- | --- | --- | --- | --- | --- | --- | --- | --- |
| *Acanthopanax senticosus* (Rupr. Et Maxim.)Harms | Araliaceae | *ciwujia* | Herb | Bark | against rheumatism, against pain, Circulatory system disorders(CIR), Muscular-skeletal system disorders (MUS) | HGDB | **1** | NB158 |
| *Aconitum carmichaeli* Debx. | Ranunculaceae | *fuzi* | Herb | Roots | Digestive system disorders(DIG), against gynecological diseases (REN) | HGDB,HGDN,FGDB,FGDN | **18** | NB15 |
| *Aconitum vilmorinianum* Kom*.* | Runaunculaceae | *dacaowu* | Herb | Roots | against rheumatism, Circulatory system disorders(CIR), against pain,poisonings | HGDN | **1** | 82209 |
| *Acorus calamus* L. | Araceae | *changpu* | Herb | Root and stem | Circulatory system disorders(CIR), inflammation, Genitourinary system disorders(GEN), | HGDB,HGDN,FGDN | **3** | 82009 |
| *Acorus gramineus* Soland. Var. Pusillus Engl. | Araceae | *xiyechangpu* | Herb | Roots and stems | Circulatory system disorders(CIR), Respiratory systems disorder(RES), inflammation, Digestive system disorder(DIG) | HGDB | **1** | NB166 |
| *Psammosilene tunicoides* W.C. Wu et C.Y.Wu | Caryophyllaceae | *dudingzi* | Herb | Roots | Nutritional disorders (NUT), against cold and flu. | HGDB | **1** | 81711 |
| *Agastache rugosa* (Fisch.et Mey.)O.Ktze | Labiatae | *huoxiang* | Herb | Whole plant | RES, DIG, inflammation | FGDN | **1** | 82017 |
| *Agrimonia pilosa* Ledab. var nepalensis (D. Don) Nakai | Rosaceae | *xianhecao* | Herb | Whole plant | anti-inflammation, DIG | HGDN | **1** | 1207021 |
| *Angelica dahurica (*Fisch. Ex Hoffm.) Benth. | Umbelliferae | *baizhi* | Herb | Roots | against rheumatism, CIR, against pain, edema, Skin/subcutaneous cellular tissue disorder(SKI), Genitourinary system disorders(GEN) | HGDB | **1** | NB157 |
| *Angelica Sinensis* (Oliv.) Diels. | Umbelliferae | *qingui* | Herb | Roots | Nutritional disorders(NUT), CIR, GEN, against pain, Digestive system disorder(DIG) | HGDB,HGDN,FGDB,FGDN | **6** | NB125 |
| *Anisodus acutangulus* C. Chen et C.L. Chen | Solanaceae | *sanfensan* | Herb | Roots | against rheumatism, mental and nervous system disorders , against pain | HGDB,HGDN,FGDB,FGDN | **5** | 82005 |
| *Araliafargesii* Franch | Araliaceae | *duhuo* | Herb | Roots | RES, poisonings, CIR, against pain | HGDB,HGDN,FGDN | **3** | 82210 |
| *Arctium lappa* L. | Asteraceae | *niupangzi* | Herb | Roots | Reducing cholesterol in the body, detoxification and anti-inflamation, Preventing gastric cancer | HGDN,FGDN | **4** | NB145 |
| *Artemisia codonocephala* Diels. | Asteraceae | *kuhao* | Herb | Whole plant | anti-inflammation, to reduce poisonings | FDGN | **1** | NB137 |
| *Aster yunnanensis* Fr. | Asteraceae | *dengzhanhua* | Herb | Roots | warming the lungs, anti-inflammation, against coughing | FGDN | **1** | NB129 |
| *Astilbe rivularis* Buch. -Ham. ex D. Don | Saxifragaceae | *hongshengma* | Herb | Roots | CIR, against rheumatism, and pain | HGDN | **1** | 82211 |
| *Aucklandia lappa* Decne. | Compositae | *muxiang* | Herb | Roots | CIR, DIG, RES, against pain | HGDB,HGDN,FGDB,FGDN | **18** | NB124 |
| *Baphicacanthus cusia*(Nees) Bremek. | Cruciferae | *banlangen* | Herb | Roots | Anti-inflammation and detoxification , CIR | HGDB | **1** | NB160 |
| *Berberis lijiangensis* C.Y.Wu  Schneid. | Berberidaceae | *xiaopo* | Herb | Roots, stems | Anti-inflammation and detoxification , against rheumatism | HGDB | **1** | NB152 |
| *Bergenia purpurascens* (Hook.f.etThoms.)Engl. | Saxifragaceae | *yanchangpu* | Herb | Whole plant | Anti-inflammation and detoxification ， against pain，CIR，against coughing | HGDB | **1** | 82214 |
| *Bletilla sinensis* (Rolf) Schltr. | Orchidaceae | *dabaiji* | Herb | Roots | CIR, SKI | HGDN,FDGB | **3** | 12072601 |
| *Boehmeria nivea*(L.)Gaud. | Urticaceae | *yuanma* | Herb | Whole plant | inflammation, SKI, detoxification, CIR, to treating traumatic injury | HGDN | **1** | 12723 |
| *Bupleurum rockii* Wolff | Umbelliferae | *chaihu* | Herb | Whole plant | RES, to treat sore and ulcer, inflammation | SGNB | **1** | NB140 |
| *Cardiocrinum giganteum* (Wall.) Makino | Liliaceae | *baihe* | Herb | Roots | Inflammation and detoxification, against coughing, and pain | FGDN,FGDB | **4** | NB135 |
| *Chaenomeles sinensis* (Thunb) Koehne | Rosaceae | *zoupimugua* | Tree | Fruits | To treat rheumatoid arthritis, lumbocrural pain, numb, DIG, mental and nervous system disorders | HGDB,HGDN,FGDB,FGDN | **16** | NB171 |
| *Cimicifuga yunnanensis*Hsiao | Ranunculaceae | *lushengma* | herb | Whole plant | CIR, inflammation, detoxification | HGDN,FGDN,HGDB | **7** | 81803 |
| *Clerodendranthus spicatus  (Thunb.) C. Y. Wu ex H. W. Li* | Labiatae | *huashicao* | Herb | Whole plant | To treat acute or chronic nephritis, and cystitis(anti-inflammation), urinary calculi(CIR), rheumatic arthritis | HGDB | **1** | NB165 |
| *Codonopsis foetens* Hook.f.& Thomson | Campanulaceae | *choudangsen* | Herb | Roots | NUT | HGDB | **1** | NB147 |
| *Codonopsis pilosula* (Franch.) Nannf. | Campanulaceae | *dangsen* | Herb | Roots | NUT | HGDB | **1** | NB168 |
| *Coptis teeta Wall.* | Ranunculaceae | *yunhuanglian* | Herb | Whole plant | inflammation, against rheumatism, detoxfication, anthelmintic(DIG) | HGDN | **1** | NB150 |
| *Coptis chinensis*Franch. | Ranunculaceae | *huanglian* | Herb | Root | to treat acute conjunctivitis(eye), DIG | HGDN | **1** | NB164 |
| *Coptis omeiensis*(Chen) C. Y. Cheng | Ranunculaceae | *chuanhuanglian* | Herb | Roots, stems | inflammation, against rheumatism, anthelmintic(DIG) | HGDN | **1** | NB151 |
| *Cyathula officinalis*Kuan | Amaranthaceae | *chuanniuxi* | Herb | Roots | CIR, NUT, to strengthen bones and tendons | HGDN | **1** | 81819 |
| *Cynanchum otophyllum* Schneid. | Asclepiadaceae | *qingyangsen* | Herb | Roots | as kidney tonic, against skin rashes, against rheumatism | FGDN,HGDN | **6** | NB122 |
| *Delphinium likiangense delavayi* Franch. | Papilionaceae | *cuique* | Herb | Roots | to drive out cold air (TCM), against rheumatism, against pain, tong luo(TCM, move vertical energy channels), CIR | FGDB | **1** | NB154 |
| *Dendranthema morifolium*(Ramat.) Tzvel. | Asteraceae | *hangbaiju* | Herb | Flowers | soothing the liver(CIR), against eye-problems, anti-inflammation, as kidney tonic(NUT), strengthening the spleen and stomach(DIG), to moist throat(RES), promoting the secretion of saliva, adjusting blood fat, RES, CIR | HGDB | **1** | NB146 |
| *Dioscorea panthaica* Prain & Burkill | Dioscoreaceae | *huashanyao* | Herb | Roots | For stomach health and indigestion (DIG) | HGDB | **1** | 1181707 |
| *Dipsacus mitis* D. Don | Dipsacaceae | *xuduan* | Herb | Roots | as liver and kidney tonic, to strengthen bones and tendons(MUS), against pain | FGDB,FGDN,HGDN | **11** | NB110 |
| *Eucommia ulmoides* | Eucommiaceae | *duzhong* | Tree | Bark | to strengthen bones and tendons(MUS), GEN, increases blood flow(CIR) | HGDB,FGDB | **5** | NB013 |
| *Eupatorium Adenophorum* Spreng | Asteraceae | *zelan* | Herb | Whole plant | against insect bites, against edema, stop bleeding (SKI) | HGDB | **1** | NB159 |
| *Eupatorium japonicum* Thunb. | Asteraceae | *xiaoceilan* | Herb | Roots | Again pain, to promote circulation(CIR), and to treat measles (SKI),treating traumatic injury(INJ) | HGDB,HGDN | **2** | NB136 |
| *Eutrema ynnanensis* Franch. | Cruciferae | *shanyucai* | Herb | Whole plant | as spice, treating cold (cold and flu) | FGDN,FGDB | **3** | NB173 |
| *Fagopyrum leptopodum*(Diels) Hedb. | Polygonaceae | *yekuqiao* | Herb | Fruits | to treat muscular–skeletal system disorders, refreshing, to treat eyes and ears disease, anti-inflammation and detoxification, CIR, DIG | HGDN,HGDB | **2** | 1207007 |
| *Fallopia multiflora* (Thunb.) Harald. | Polygonaceae | *heshouwu* | Liana | Whole plant | anti-inflammation and detoxification , Genitourinary system disorders(GEN), stop bleeding | HGDN,HGDB,FGDN,FGDB | **16** | 82007 |
| *Foeniculum vulgare* Mill | Umbelliferae | *huexian* | Herb | Roots | Anti-inflammation, moistening gallbladder(DIG), against coughing | FGDB,FGDN,HGDN,HGDB | **16** | 82006 |
| *Fritillaria cirrhosa* D. Don | Orchidaceae | *beimu* | Herb | Roots | moistening lung, anti-inflammation, against coughing | HGDN | **1** | NB162 |
| *Gastrodia elata var.elata* | Orchidaceae | *tianma* | Herb | Roots | soothing the liver(RES), to drive out cold air of body (TCM), stop spasm, against headache and dizziness, numbness of the limbs, convulsion on children, spasm of epilepsy, tetanus (mental and nervous system disorders) | HGDN | **1** | NB144 |
| *Gentiana robusta* King ex Hook. f. | Geraniaceae | *qinjiao* | Herb | Whole plant | anti-inflammation | FGDB,FGDN | **16** | NB148 |
| *Geum aleppicum* Jacq. | Umbelliferae | *longxucao* | Herb | Whole plant | Anti-inflammation and detonicficaiton, against edema, against pain, against rheumatism | HGDB | **1** | NB133 |
| *Goodyera schlechtendaliana* Rchb.f | Liliaceae | *xiaojiangjun* | Herb | Whole plant | against mycosis | HGDB | **1** | NB130 |
| *Gymnadenia orchidis*Lindl. | Orchidaceae | *fuzhangsen* | Herb | Roots | as kidney tonic(NUT), stimulating sperms(GEN) , RES, against pain | HGDN | **1** | NB161 |
| *Gynura japonica* (L.f.) Juel | Asteraceae | *tusanqi* | Herb | Roots | CIR,GEN, stop bleeding, anti-inflammation and detoxification | HGDB,HGDN,FGDN,FGDB | **6** | NB149 |
| *Gynura japonica* (Linn.f.) Juel | Asteraceae | *niutouqi* | Herb | Whole plant | CIR, stop bleeding, mental and nervous system disorders | HGDN | **1** | NB170 |
| *Hemsleya macrosperma* C.Y.Wu | Cucurbitaceae | *luoguodi* | Herb | Roots | anti-inflammation, detoxification, against coughing, eliminating phlegm | FGDB | **1** | NB11 |
| *Hemsleya lijiangensis*A. M. Lu ex C. Y. Wu et C. L. Chen | Cucurbitaceae | *xuedan* | Herb | Roots | anti-inflammation, detoxification,, DIG, against pain | FGDB | **1** | NB163 |
| *Herba Salviae* Plbeiae | Labiatae | *lizhicao* | Herb | Whole plant | anti-inflammation, detoxification, cooling blood, as a diuretic | HGDB | **1** | NB121 |
| *Houttuynia cordata* Thunb. | Saururaceae | *yuxingcao* | Herb | Whole plant | anti-inflammation, detoxification, to treat sore and ulcer(SKI), and fester, diuretic, treating stranguria (CIR) | FGDB,FGDN | **3** | NB19 |
| *Hylotelephium verticillatum* (Linn.) H. Ohba | Crassulaceae | *yansanqi* | Herb | Whole plant | stop bleeding, against pain | HGDN | **1** | NB143 |
| *Jasminum nudiflorum Lindl.* | Oleaceae | *yingchunhua* | Shrub | Branch | Against fever, headache, against pharyngitis (RES), swelling and pain in throat, cough with lung heat, GEN, abscess, treating traumatic injury and stop bleeding (INJ and SKI) | HGDB, FGDB | **2** | NB174 |
| *Lactuca sativa* L. | Asteraceae | *wosun* | Herb | Stem | GEN, CIR, swelling and pain, treating traumatic injury, fracture (MUS) | FGDN,FGDB | **18** | NB177 |
| *Ledebouriella divaricata* (Turcz.) Hiroe | Umbelliferae | *zhuyefangfeng* | Herb | Whole plant | RES, anti-inflammation, fever, to treat sore and ulcer (SKI) | HGDB | **1** | NB15 |
| *Leycesteria formosa* Wall. | Caprifoliaceae | *meiyeizhu* | Herb | Whole plant | anti-inflammation and detoxification , against pain and CIR | HGDB | **1** | NB169 |
| *Ligusticum chuanxiong* S.H.Qiu | Umbelliferae | *chuanxiong* | Herb | Roots | purging and to dispel stasis, to drive out cold air (TCM), cooling blood, to increases blood flow and against pain, to treat headache and dizziness, rib and abdominal pain, amenorrhea, dystocia, sore and ulcer | FGDN,FGDB,HGDB,HGDN | **14** | NB111 |
| *Magnolia officinalis* Rehd.et Wils | Magnoliaceae | *houpu* | Tree | Bark | treating a lump in the abdomen and distending pain, gastric disorder causing nausea, vomit, dyspepsia, phlegm and fluid retention dyspnea with cough, cold-dampness and diarrhea | HGDB | **1** | NB12 |
| *Mahonia hancockiana Takeda* | Berberidaceae | *shidagonglao* | Herb | Stems roots | anti-inflammation , detoxification | HGDB | **1** | NB19 |
| *Mentha spicata* L. | Labiatae | *buhuo* | Herb | Leave | Promote body circulation, against pain, stop coughing | FGDB,FGDN | **14** | 82016 |
| *Notopterygium incisum* Ting ex H. T. Chang | Umbelliferae | *qianghuo* | herb | Roots | against rheumatism, increases blood flow, against pain, against edema | HGDB,HGDN,FGDN | **4** | 81810 |
| *Paeonia delavayi* Franch. | Ranunculaceae | *yemudan* | Herb | Root-bark | as blood tonic, soothing the liver, to reduce sweating, against pain | HGDB | **1** | NB167 |
| *Paeonia delavayi* Franch. Var. lutea(Delav. Ex Franch.) Finet. Et Gagnep. | Ranunculaceae | *mudan* | Herb | Root-bark | anti-inflammation and detoxification, against pain, to treat hematuria, against menstruation pain | HGDB,HGDN,FGNB,FGND | **15** | NB13 |
| *Paris polyphylla Smith var yunnanensis* (Fr.) Hand.-Mazz. | Trilliaceae | *chonglou* | Herb | Roots | anti-inflammation and detoxification ，cooling blood | HGDB,FGDB,HGDN,FGDN | **16** | NB014 |
| *Phlegmariurus austrosinicus* (Ching) L. B. Zhang | Huperziaceae | *dabusi* | Herb | Whole plant | against edema, against pain, anti-inflammation and detoxification | HGDN,FGDN | **2** | NB155 |
| *Phytolacca acinosa* Roxb. | Phytolaccaceae | *shanglu* | Herb | Roots | unblocking urinary passage, against coughing and eliminating phlegm , reducing swelling, against edema, diuretic and against constipation, against pharyngitis, swelling and pain in throat, external skin treatment abscess | HGDB,HGDN,FGDB | **5** | 81826 |
| *Pimpinella candolleana* Wight et Arn. | Umbelliferae | *xingyefangfeng* | Herb | Whole plant | Diaphoresis, purging, against rheumatism, to increases blood flow, against edema | HGDB | **1** | NB132 |
| *Plantago schneideri* Pilger | Plantaginaceae | *cheqiancao* | Herb | Whole plant | anti-inflammation and detoxification , diuretic, against coughing, against eye-problems | FGDB | **2** | NB128 |
| *Platycladus orientalis* (Linn.) Franch. | Cupressaceae | *cebai* | Tree | Stems | anti-inflammation , diuretic, moistening lung, against coughing, stimulating hair growth, hair-blacking | FGDN,HGDN | **3** | 12081601 |
| *Platycodon grandiflorus* (Jacq.) A.DC. | Campanulaceae | *jigeng* | Herb | Roots | against coughing, eliminating phlegm, anti-inflammation, to treat pleurisy | FGDN,FGDB,HGDB,HGDN | **15** | NB14 |
| *Polygala arillata* Buch-Ham ex D.Don | Polygalaceae | *hebaoshanguihua* | Tree | Whole plant | strengthening the stomach, regulating irregular and too rare menstruation, against edema | HGDB,HGDN | **3** | NB172 |
| *Polygonatum cirrhifolium* (Wall.) Royle | Liliaceae | *huangjing* | Herb | Roots | promoting the secretion of saliva, moistening lung, invigorating spleen, as kidney tonic, release toxins | FGDB | **2** | 1182225 |
| *Polygonum amplexicaule* D. Don. | Polygonaceae | *yantuo* | Herb | Roots | against rheumatism, against pain | HGDB,HGDN | **4** | NB141 |
| *Reynoutria joponica* Houtt. | Polygonaceae | *huzhang* | Herb | Whole plant | to drive out cold air (TCM), diuretic, shu jing (TCM, stretch tendons) and to dispel stasis. to treat jaundice, gallstone, dysentery, constipation, leucorrhea, amenorrhea, arthralgia, scald, eczema, herpes zoster, furuncle, Pyogenic infection, against contusions | HGDN | **1** | 81827 |
| *Polygonum paleaceum* Wall. | Polygonaceae | *xuejie* | Herb | Whole plant | clear heat and release toxins, unblocking urinary passage, to dispel stasis, against eye-problems | HGDB, HGDN, FGDN | **7** | NB17 |
| *Potentilla peduncularis* D. Don | Rosaceae | *diyu* | Herb | Roots | to stimulate digestion, anti-diarrhea effect(DIG), anti-inflammatio, stop bleeding | HGDN | **1** | 1207022 |
| *Prinsepia utilis* Role | Rosaceae | *qingci* | Shrub | Fruits | anti-inflammation and detoxification, to dispel stasis and against pain(CIR) | FGDN,FGDB | **11** | 81805 |
| *Paeonia lactiflora* Pall. | Paeoniaceae | *shuoyao* | Herb | Whole plant | nourishing blood and liver(NUT), against pain, anti-inflammation | FGDN,FGDB | **5** | NB016 |
| *Reineckia carnea* (Andr.) Kunth | Liliaceae | *jixiangcao* | Herb | Whole plant | moistening lung(RES), against coughing, to drive out cold air of body(CIR), to treat broken bones(MUS) | HGDB | **1** | NB018 |
| *Rheum nepalensis* Spreng | Polygonaceae | *tudaohuan* | Herb | Roots | CIR, to reduce bad blood, anti-inflammation | FGDN,HGDN | **10** | 82208 |
| *Atractylodes macrocephala* | Compositae | *baishu* | Herb | Stems, Roots | NUT ,GEN, anti-inflammation, PRE | FGDB | **9** | NB18 |
| *Rhus chinensis* Mill. | Schisandraceae | *wubeizi* | Herb | Sap | stop bleeding, anti-inflammation | HGDB | **1** | NB138 |
| *Rodgersia aesculifolia batal* | Saxifragaceae | *guidengqing* | Herb | Roots and stems | anti-inflammation and detoxification , cooling blood, metabolic system disorder | HGDN | **1** | 81815 |
| *Rheum palmatum* L. | Polygonaceae | *dahuang* | Herb | Roots | anti-inflammation and detoxification , purgation, anthelmintic(DIG), stop bleeding, GEN | HGDB FGDB  FGDN | **16** | NB127 |
| *Salvia przewalskii* Maxim. | Labiatae | *zidansen* | Herb | Roots | CIR, GEN | FGDB | **2** | NB017 |
| *Sambucus adnata*Wall. ex DC. | Caprifoliaceae | *xuemangcao* | Herb | Whole plant | CIR, to dispel stasis, against rheumatism, diuretic | FGDN | **1** | NB156 |
| *Sargentodoxa cuneata*(Oliv.) Rehd. et Wils. | Sargentodoxaceae | *tiegusan* | Liana | Roots and stems | CIR, to dispel stasis, against pain, RES, anthelmintic (DIG) | HGDB | **1** | NB138 |
| *Schizonepeta tenuifolia(Benth.)* Briq. | Labiatae | *jingjie* | Herb | Whole plant | Anti-inflammation, and relieving pains, colds, against skin rashes, to treat sore and ulcer, stop bleeding (SKI) | HGDB,FGDB | **3** | NB012 |
| *Sophora flavescens* Aiton | Fabaceae | *kusen* | Srub | Roots | anti-inflammation and detoxification, anti-tumor, against heart disorders | HGDN,FGDN | **6** | 1207057 |
| *Stemona japonica*(Bl.) Miq | Ruscaceae | *baibu* | Herb | Roots | CIR, against coughing, coughing | HGDN | **1** | 82410 |
| *Talinum trianglare*（Jacy.）Willd. | Arliaceae | *tugaolisen* | Herb | Roots | anti-inflammation and detoxification | HGDB | **1** | NB0126 |
| *Taraxacum dissectum* (Ledeb.) Ledeb. | Ateraceae | *pugongying* | Herb | Whole plant | anti-inflammation and detoxification , against edema | FGDN,FGDB,HGDN | **3** | NB0134 |
| *Taxus yunnanensis* W.C. Cheng & L.K.Fu | Taxaceae | *hongdousha* | Tree | Branch | DIG, indigestion | FGDN | **3** | NB0175 |
| *Thalictrum glandulosissimum* (Finet.et Gagnep.) W.T.Wang et S.H.Wang | Ranunculaceae | *maweihuanglian* | Herb | Roots | anti-inflammation, to drive out cold air of body, CIR, To cure children’s fever, cold, and rash | HGDB | **1** | NB016 |
| *Toona sinensis* Roem | Meliaceae | *yexiangchun* | Tree | Leave | anti-inflammation, deprive the evil wetness, against diarrhoea , stop bleeding, anthelmintic (DIG) | FGDN,HGDN | **2** | 82221 |
| *Verbascum thapsus* L. | Scrophulariaceae | *yizhuxiang* | Herb | Whole plant | anti-inflammation and detoxification , stop bleeding, CIR | HGDB | **1** | NB0120 |
| *Viola japonica* Langsd. | Violaceae | *litoucao* | Herb | Whole plant | clear heat and release toxins, removing fester, anti-inflammation | HGDB | **1** | NB0131 |
| *Zanthoxylum bungenum* maxim | Rutaceae | *huajiao* | Tree | Fruits | Against cold-dampness, diarrhoea and dysentery(DIG), pruritus(SKI), boils on the mouth and tongue, against drunkenness | FGDN, FGDB | **12** | NB0176 |
| *Zingiber officinarum* Hance | Zingiberaceae | *yeshengjiang* | Herb | Roots | CIR,GEN, against coughing, and edema, detoxification | FGDN,HGDN | **4** | NB0142 |

**Note: Healer’s Garden (HG), Farmer's Garden (FG), Diannan village (DN), Dianbei village (DB); HGDN (Healers’s homegarden in Diannan village); HGDB (Healers’s homegarden garden in Dianbei village); FGDN (Farmers’ homegarden in Diannan village); FGDB (Farmer’s homegarden in Dianbei village)**
